# Clinical Characteristics and Risk Factors of Pyogenic Spondylitis Caused by Gram-Negative Bacteria

**DOI:** 10.1371/journal.pone.0127126

**Published:** 2015-05-15

**Authors:** Seung-Ji Kang, Hee-Chang Jang, Sook-In Jung, Pyoeng Gyun Choe, Wan Beom Park, Chung-Jong Kim, Kyoung-Ho Song, Eu Suk Kim, Hong Bin Kim, Myoung-don Oh, Nam Joong Kim, Kyung-Hwa Park

**Affiliations:** 1 Department of Infectious Diseases, Chonnam National University Medical School, Gwangju, South Korea; 2 Department of Internal Medicine, Seoul National University College of Medicine, Seoul, South Korea; Vanderbilt University, UNITED STATES

## Abstract

**Background:**

There are limited data describing the clinical characteristics of pyogenic spondylitis caused by Gram-negative bacteria (GNB). The aim of this study was to investigate the predisposing factors and clinical characteristics of pyogenic spondylitis caused by GNB compared to Gram-positive cocci (GPC).

**Methods:**

We performed a retrospective review of medical records from patients with culture-confirmed pyogenic spondylitis at four tertiary teaching hospitals over an 8-year period.

**Results:**

A total of 344 patients with culture-confirmed pyogenic spondylitis were evaluated. There were 62 patients (18.0%) with pyogenic spondylitis caused by GNB and the most common organism was *Escherichia coli* (n = 35, 10.2%), followed by *Pseudomonas aeruginosa* (n = 10, 2.9%). Pyogenic spondylitis caused by GNB was more frequently associated with the female gender (64.5 vs. 35.5%, *P* <0.01), preexisting or synchronous genitourinary tract infection (32.3 vs. 2.1%, *P*< 0.01), and intra-abdominal infection (12.9 vs. 0.4%, *P*< 0.01) compared to patients with GPC. Although pyogenic spondylitis caused by GNB presented with severe sepsis more frequently (24.2 vs. 11.3%, *P* = 0.01), the mortality rate (6.0 vs. 5.2%) and the proportion of patients with residual disability (6.0 vs. 9.0%), defined as grade 3 or 4 (*P* = 0.78) 3 months after completion of treatment, were not significantly different compared to GPC patients.

**Conclusion:**

GNB should be considered as the etiologic organism when infectious spondylitis develops in a patient with preexisting or synchronous genitourinary tract and intra-abdominal infection. In addition, the mortality rate and clinical outcomes are not significantly different between pyogenic spondylitis caused by GNB and GPC.

## Introduction

Infectious spondylitis is a destructive infection of the spine or paraspinal structures [1,2]. As spondylitis progresses, vertebrae are destroyed, and inflammation extends to the epidural and paraspinal spaces. The most detrimental consequence of infectious spondylitis is neurologic deficits, which develop in approximately one-third of cases.

Spinal infections can be described etiologically as pyogenic, granulomatous (tuberculous, brucella, fungal) and parasitic [3]. Pyogenic spondylitis refers to infectious spondylitis caused by bacteria; *Staphylococcus aureus* and streptococci are the most common microorganism encountered. The proportion of Gram-negative bacteria (GNB) varies between studies, constituting 15–39% of the etiologic microorganisms causing pyogenic spondylitis [4–7].

Microbiologic diagnosis is an important step in the management of pyogenic spondylitis, because it requires long-term antibiotic treatment. However, despite various efforts, in some patients, etiologic organisms are not isolated and antibiotics are chosen empirically. The most commonly used empirical antibiotics are first-generation cephalosporins, which are active against methicillin-susceptible *S*. *aureus* and streptococci [8]. The antibiotic susceptibilities of GNB vary between species, and are frequently not susceptible to first-generation cephalosporins. Predictive factors for GNB in patients with pyogenic spondylitis are critical for choosing empirical antibiotics. However, few studies have identified the predisposing factors or clinical characteristics of pyogenic spondylitis caused by GNB. Moreover, studies of GNB pyogenic spondylitis report results from less than 10 patients, with the exception of one study that reported data from 65 patients [9].

The objectives of this study were to investigate the predisposing factors for GNB in patients with pyogenic spondylitis and the clinical characteristics of pyogenic spondylitis caused by GNB.

## Methods

### Study design

We conducted a retrospective cohort study at four university-affiliated teaching hospitals from January 2005–March 2013. We retrospectively collected medical records of patients ≥ 18 years of age with pyogenic spondylitis. Only culture-confirmed patients with pyogenic spondylitis were included, and patients with infectious spondylitis caused by *Brucella* species, *M*. *tuberculosis*, or fungi polymicrobial infection were excluded. We collected demographic data, co-morbidities, presenting symptoms, microbiologic data, radiographic characteristics, and laboratory data including white blood cell (WBC) counts, C-reactive protein (CRP), surgical treatment, clinical outcomes, and mortality rate. The presence of the following predisposing factors was also documented: preexisting or synchronous infection, presence of spinal prosthesis, spinal surgery within 1 year, and previous invasive spinal procedures within 6 months, such as epidural block, acupuncture and percutaneous vertebroplasty. Clinical outcomes of mortality and neurologic outcome were evaluated at 3 months and 12 months after completion of treatment. To investigate predisposing factors and clinical characteristics, we compared GNB pyogenic spondylitis with Gram-positive cocci (GPC) spondylitis. The Institutional Review Boards of the four participating hospitals (Chonnam National University Hospital Institutional Review Board, Chonnam National University Hwasun Hospital Institutional Review Board, Hwasun, Seoul National University Hospital Institutional Review Board, and the Seoul National University Bundang Hospital Institutional Review Board) approved this study. A waiver of consent was granted, given the retrospective nature of the project.

### Definition

Pyogenic spondylitis was diagnosed when the etiologic organism was isolated from spinal or paraspinal tissues, or if there were compatible clinical signs or symptoms and radiologic evidence of vertebral infection, and positive blood cultures which were performed at the time of diagnosis of pyogenic spondylitis, including at least two separate sets of blood cultures in the case of common skin contaminants, such as coagulase-negative staphylococci. Compatible signs or symptoms of vertebral infection were defined as pain, fever, or neurologic manifestations. Typical MRI characteristics included decreased signal intensity in the vertebral body and disk and loss of end plate on T1-weighted images and increased signal intensity of the disk and vertebral body on T2-weighted images [10]. Preceding bacteremia was defined as bacteremia from any bacteria within 12 months before the diagnosis of spondylitis [4].

Preexisting or synchronous infection was defined as a documented infection at another site within the 30 days prior to or at the diagnosis of pyogenic spondylitis [10]. Severe sepsis was defined as sepsis with one or more signs of organ dysfunction. Neurologic staging was divided into five grades, which was adopted from previous report [11]; no pain (grade 0), back pain at the level of the affected spine (grade 1), nerve-root pain radiating from involved spinal area (grade 2), motor weakness, sensory deficit, or bladder/bowel dysfunction (grade 3), and paralysis (grade 4). Outcomes were evaluated using overall mortality, duration of treatment, and residual disability. Residual disability was defined as grade 3 or 4 by neurologic staging.

### Statistical analysis

The Student *t-*test or Mann-Whitney U-test was used to compare continuous variables. The Chi-square test or Fisher’s exact test was used to compare categorical variables. A logistic regression model was adopted to adjust for confounding variables and to identify risk factors. To this end, we performed a stepwise multivariate logistic regression analysis. Variables with *P* < 0.10 using univariate analysis were candidates for multivariate analysis. The odds ratio (OR) and 95% confidential intervals (CIs) were calculated. All *P* values were two-tailed, and *P* < 0.05 was considered to indicate statistical significance. Statistical analysis was performed using the SPSS software (version 20.0; SPSS Inc., Chicago, IL, USA).

## Results

### Etiologic microorganisms

During the study period, a total of 659 patients with infectious spondylitis were identified. Spinal or paraspinal tissue biopsy was performed in 522/659 cases (79.2% of total spondylitis during this study), and microorganisms were not identified in 24.1%. Among them, 344 patients were diagnosed with culture-confirmed pyogenic spondylitis, 282 cases (82.0%) were caused by GPC, and 62 cases (18.0%) were caused by GNB (Table 1). The most common GPC isolate was *S*. *aureus* (n = 163, 47.5%) followed by streptococcal species (n = 59, 17.2%), and coagulase-negative staphylococci (n = 51, 14.8%). The common GNB were *Escherichia coli* (n = 35, 10.2%), *Pseudomonas aeruginosa* (n = 10, 2.9%), and *Klebsiella pneumoniae* (n = 8, 2.3%). Blood cultures were obtained initially in 249 patients (88.3%) in the GPC group and 54 patients (87.1%) in the GNB group. Positive blood cultures were more often identified in 198 (79.5%) patients in the GPC group and 34 (63.0%) patients in the GNB group (*P* = 0.01). Tissue cultures were completed for 205 (72.7%) and 51 (82.3%) patients in each group, and of these, 171 (83.4%) and 43 (84.3%) patients showed positive tissue culture results, respectively. In 87 patients (30.9%) of GPC group and 15 patients (24.2%) of GNB group, the etiologic organism was isolated from both tissue and blood. Antibiotic exposures prior to blood culture were observed in 25 (10.0%) and 7 (13.0%) of patients with Gram-positive and -negative bacteria. Antibiotic exposures prior to tissue biopsy were observed in 83 (40.5%) and 31 (60.8%) patients in the two groups, respectively.

**Table 1:** Causative organisms identified in 344 patients with pyogenic spondylitis.

Microorganism	344 (%)
**Gram-positive Cocci**	
*Staphylococcus* spp.	214 (62.2)
Methicillin-susceptible *S*. *aureus*	96 (27.9)
Methicillin-resistant *S*. *aureus*	67 (19.5)
Methicillin-susceptible coagulase-negative staphylococci	10 (2.9)
Methicillin-resistant coagulase-negative staphylococci	41 (11.9)
*Streptococcus* spp.	59 (17.2)
*S*. *agalactiae*	12 (3.5)
viridans streptococci	37 (10.8)
*S*. *pneumoniae*	3 (0.9)
Other *Streptococcus* spp.	7 (2.0)
*Enterococcus* spp.	9 (2.6)
**Gram-negative Bacteria**	
*Escherichia coli*	35 (10.2)
*Pseudomonas aeruginosa*	10 (2.9)
*Klebsiella pneumoniae*	8 (2.3)
*Serratia marcescens*	3 (0.9)
*Enterobacter cloacae*	2 (0.6)
*Klebsiella oxytoca*	1 (0.3)
*Serratia rubidaea*	1 (0.3)
*Citrobacter koseri*	1 (0.3)
Salmonella serogroup B	1 (0.3)

### Antibiotic susceptibilities of GNB

The antibiotic susceptibilities of GNB causing pyogenic spondylitis are summarized in Table 2. Of 52 GNB isolates belonging to the *Enterobacteriaceae* family, 30.8% were resistant to fluoroquinolone, and 15.4% to third-generation cephalosporins. We identified 2 isolates from a total of 10 *P*. *aeruginosa* samples that were resistant to fluoroquinolone and 3 isolates that were resistant to anti-pseudomonal third-generation cephalosporins. The resistance rates to fluoroquinolone or third generation cephalosporins were not different in patients with or without previous spinal surgery or an invasive spinal procedure.

**Table 2:** Antibiotic susceptibilities of Gram-negative bacteria causing pyogenic spondylitis.

	Without previous surgery nor invasive spinal procedures ^a^			With previous surgery or invasive spinal procedures ^a^		
Species (No.)	Resistant to ciprofloxacin n/No.	Resistance to 3^rd^ generation cephalosporin (ceftazidime in *Pseudomonas aeruginosa*) n/No.	Resistance to carbapenem n/No.	Resistant to ciprofloxacin n/No.	Resistant to 3rd generation cephalosporin (ceftazidime in *Pseudomonas aeruginosa* n/No.	Resistance to carbapenem n/No.
*E*. *coli* (35)	12/25	4/25	0/25	3/10	2/10	0/10
*P*. *aeruginosa* (10)	2/5	2/5	2/5	0/5	1/5	0/5
*K*. *pneumoniae* (8)	0/4	0/4	0/4	1/4	2/4	0/4
*S*. *marcescens* (3)	0/2	0/2	0/2	0/1	0/1	0/1
*E*. *cloacae* (2)	0/1	0/1	0/1	0/1	0/1	0/1
*C*. *koseri* (1)	0/1	0/1	0/1			
*K*. *oxytoca* (1)	0/1	0/1	0/1			
Salmonella (1)	0/1	0/1	0/1			
*S*. *rubidaea* (1)				0/1	0/1	0/1

^a^ Previous invasive spinal procedures: epidural block, spinal prosthesis, acupuncture and percutaneous vertebroplasty within 6 months before diagnosis of pyogenic spondylitis

### Clinical and radiographic characteristics of pyogenic spondylitis caused by GNB

The demographic findings and clinical characteristics of pyogenic spondylitis caused by GNB and GPC are shown in Table 3. The mean age (± SD) was similar in both groups. Female gender was more frequently associated with GNB infection (64.5 vs. 35.5%, *P*< 0.01). The most frequently reported symptom at diagnosis was pain at the affected site in each group. Fever and severe sepsis were observed more frequently in the GNB group (67.7 vs. 53.2%, *P* = 0.05; 24.2 vs. 11.3%, *P* = 0.01, respectively). The most frequently involved vertebral level was the lumbar spine in both groups, and no difference was observed in the involved vertebral level or paraspinal involvement between the two groups. The proportion of patients initially presenting with grade 3 or 4 was not different (16.4 vs. 18.2%, *P* = 0.85) between the GNB and GPC groups.

**Table 3:** Demographics and clinical characteristics of 344 patients with pyogenic spondylitis.

	GNB, 62 (%)	GPC, 282 (%)	*P*-value
**Patient demographics**			
Age, mean ± SD	65.2 ± 12.0	63.5 ±12.0	0.30
Female	40 (64.5)	100 (35.5)	<0.01
Co-morbidity			
Diabetes mellitus	16 (25.8)	91 (32.3)	0.37
Liver cirrhosis	10 (16.1)	24 (8.5)	0.10
Chronic kidney disease	6 (9.7)	29 (10.3)	>0.99
Renal replacement therapy	2 (3.2)	11(3.9)	>0.99
Congestive heart failure	2 (3.2)	10 (3.5)	>0.99
Solid tumor	8 (12.9)	24 (8.5)	0.33
Hematologic malignancy	2 (3.2)	4 (1.4)	0.30
Pain on affected site	58 (93.5)	268 (95.0)	0.54
Duration of Pain (median days, range)	15 (1–180)	10 (0–720)	0.88
Fever	42 (67.7)	150 (53.2)	0.05
Sepsis	32 (51.6)	145 (51.4)	>0.99
Severe sepsis	15 (24.2)	32 (11.3)	0.01
**Initial Neurologic staging** ^a^	61(%)	274(%)	
Grade 0	2 (3.3)	8 (2.9)	>0.99
Grade 1	32 (52.5)	117 (42.7)	0.17
Grade 2	17 (27.9)	99 (36.1)	0.22
Grade 3	10 (16.4)	37 (13.5)	0.56
Grade 4	0 (0)	13 (4.7)	0.14
**Laboratory data**			
WBC (/mm^3^, mean ± SD)	11,224 ± 6,663	12,2178 ± 5,731	0.23
C-reactive protein (mg/dL, mean ± SD)	12.4 ± 9.1	13.2 ± 9.9	0.60
Positive blood culture (%)	34/54 (63.0)	198/249 (79.5)	0.01
Positive tissue culture (%)	43/51 (84.3)	171/205 (83.4)	>0.99
**Antibiotics Exposure**			
Prior to blood culture	7/54 (13.0)	25/249 (10.0)	0.48
Prior to tissue culture	31/51 (60.8)	83/205 (40.5)	0.01
**Radiographic findings**			
Involved spine			
C-spine	3 (4.8)	27 (9.6)	0.32
T-spine	9 (14.5)	39 (13.8)	0.84
L-spine	39 (62.9)	157 (55.7)	0.32
C-T-spine	1 (1.6)	2 (0.7)	0.45
T-L-spine	4 (6.5)	18 (6.4)	>0.99
L-S-spine	6 (9.7)	39 (13.8)	0.53
Number of affected spine (mean± SD)	2.1 ± 0.4	2.2 ± 1.0	0.45
Epidural abscess	28 (45.2)	154 (54.6)	0.21
Paraspinal abscess	30 (48.4)	147 (52.1)	0.67
Psoas abscess	23 (37.1)	75 (26.6)	0.12
**Previous invasive spinal procedures**			
Epidural block within 6 months	4 (6.5)	46 (16.3)	0.05
Vertebroplasty within 6 months	3 (4.8)	11 (3.9)	0.72
Spinal prosthesis	1 (1.6)	22 (7.8)	0.09
Acupuncture within 6 months	12 (19.4)	67 (23.8)	0.51
**Spinal surgery within 1 year**	10 (16.1)	50 (17.7)	0.86
**Mortality at 3 months after treatment** ^b^	3/50 (6.0)	12/233 (5.2)	0.73
**Neurologic staging at 3 months after treatment** ^b^	50 (%)	233 (%)	
Grade 0	22 (44.0)	92 (39.5)	0.61
Grade 1	15 (30.3)	72 (30.9)	0.90
Grade 2	7 (14.0)	36 (15.5)	0.79
Grade 3	2 (4.0)	12 (5.2)	>0.99
Grade 4	1 (2.0)	9 (3.9)	>0.99

C, cervical; GNB, Gram-negative bacteria; GPC, Gram-positive cocci; L, lumbar; S, sacrum; SD, standard deviation; T, thoracic; WBC, white blood cell count

^a^ In one case of GNB and eight of the GPC group patients, the neurologic staging was not evaluable due to other underlying neurologic disease

^b^ In eight case of GNB and twenty nine of the GPC group patients, mortality and the neurologic staging were not evaluable due to transfer to another hospital during treatment

### Predisposing factors for pyogenic spondylitis caused by GNB

A preexisting, defined as within the 30 days prior to admission, or synchronous non-spinal infection was documented in 98 (28.5%) patients and more frequently in GNB spondylitis (50.0 vs. 23.8%, *P*< 0.01; Table 4). Genitourinary tract infection (32.3 vs. 2.1%, *P*< 0.01) and intra-abdominal infection (12.9 vs. 0.4%, *P*< 0.01) were also more common in the GNB group. In contrast, skin and soft tissue infection, postoperative wound infection, and infective endocarditis were more common in the GPC group, albeit not significantly so. A history of preceding epidural block within 6 months was reported more frequently in the GPC group than in the GNB group (6.5 vs. 16.3%, *P* = 0.05). The proportion of patients who had previous invasive spinal procedures, other than epidural block, was not different between the GNB and GPC groups (Table 3). In multivariate analysis, female gender, preexisting or synchronous genitourinary tract, and intra-abdominal infection were found to be significantly associated with GNB infection (Table 5).

**Table 4:** Preexisting or synchronous non-spinal infection in 344 patients with pyogenic spondylitis.

	GNB 62 (%)	GPC 282 (%)	*P*-value
Preexisting or synchronous non spinal infection	31 (50.0)	67 (23.8)	<0.01
Skin and soft tissue infection	0 (0)	15 (5.3)	0.08
Infective endocarditis	0 (0)	14 (5.0)	0.08
Catheter related infection	1 (1.6)	8 (2.8)	>0.99
Post-operative wound infection (non-spinal operation)	0 (0)	6 (2.1)	0.60
Pneumonia	2 (3.2)	4 (1.4)	0.30
Arthritis	0 (0)	4 (1.4)	>0.99
A-V fistula infection	0 (0)	2 (0.7)	> 0.99
Deep neck infection	0 (0)	1 (0.4)	> 0.99
Meningitis	0 (0)	1 (0.4)	> 0.99
Genitourinary tract infection	20 (32.3)	6 (2.1)	< 0.01
Intra-abdominal infection	8 (12.9)	1 (0.4)	< 0.01
Others ^a^	0 (0)	5 (1.8)	0.59
Previous bacteremia within 1 year	10 (16.1)	23 (8.2)	0.06

GNB, Gram-negative bacteria; GPC, Gram-positive cocci

^a^ One endovascular stent infection, one endophthalmitis, three primary bacteremia

**Table 5:** Factors associated with GNB infection in multivariate analysis.

Factor	Univariate analysis			Multivariate analysis		
	OR	95% CI	*P*	aOR	95% CI	*P*
Female	3.31	1.86–5.88	<0.01	3.02	1.50–6.10	<0.01
Epidural block within 6 months	0.35	0.12–1.02	0.05	0.35	0.10–1.18	0.09
Spinal prosthesis	0.19	0.03–1.47	0.09	0.26	0.03–2.10	0.21
Previous bacteremia within 1 year	2.17	0.97–4.82	0.06	0.79	0.24–2.62	0.70
Fever	1.85	1.03–3.31	0.05	1.28	0.62–2.61	0.51
Severe sepsis	2.49	1.25–4.96	0.01	1.33	0.52–3.39	0.55
Preexisting or synchronous genitourinary tract infection	21.9	8.32–57.69	<0.01	19.23	6.51–56.83	<0.01
Preexisting or synchronous Intra-abdominal infection	41.63	5.1–339.67	<0.01	71.38	7.35–693.55	<0.01

CI, confidence interval; GNB, Gram-negative bacteria; OR, odds ratio

### Clinical outcomes

The most frequent antibiotics used in patients with GNB pyogenic spondylitis were third generation cephalosporins (40.3%), followed by carbapenem (25.8%), and quinolone (21.0%). Thirty-five (56.5%) patients in the GNB group and 147 patients (52.1%) in the GPC group underwent surgical treatment (*P* = 0.58). The most common surgical treatment in patients with pyogenic spondylitis caused by GNB was a simple abscess drainage with irrigation (51.4%, 18/35), followed by neurologic complications defined by grade 3 or 4 (20%, 7/35), intractable pain (11.4%, 4/35), surgical biopsy (11.4%, 4/35), and spinal instability (5.7%, 2/35). While the most common surgical treatment in patients with pyogenic spondylitis caused by GPC was a simple abscess drainage with irrigation (53.1%, 78/147), followed by neurologic complications defined by grade 3 or 4 (27.9%, 41/147), spinal instability (6.8%, 10/147), intractable pain (6.1%, 9/147), and surgical biopsy (6.1%, 9/147). Forty-six patients from both the GNB and GPC groups (GNB: 8/35 (22.9%); GPC 38/147 (25.9%)) had undergone surgical treatment two or more times for the following reasons: spinal fusion or instrumentation in 24 patients (52.2%), additional decompression or abscess drainage in 13 patients (28.3%), post-op complications such as wound infection, or screw migration in 7 (15.7%), and screw removal in 2 (4.3%) patients. The median duration of treatment for patients with GNB spondylitis was 58 days (19–415 days). Sixty-one patients (17.7%) could not be evaluated because they were transferred to another hospital during treatment. The overall mortality rate was 6.0% and 5.2% in each group at 3 months (*P* = 0.73), and 6.7% and 7.3% in each group at 12 months (*P*>0.99). The neurologic outcome was evaluated in 283 patients 3 months after treatment and 6.0% (3/50) of patients in the GNB group and 9.0% (21/233) of patients in the GPC group had residual disability defined as grade 3 or 4 (*P* = 0.78).

## Discussion

In the current study, 344 patients were microbiologically diagnosed with pyogenic infectious spondylitis. The proportion of patients with GNB was 18.0%, and the most common bacterium was *E*. *coli*, followed by *P*. *aeruginosa*, which is consistent with previous reports. Presenting symptoms, involvement of the spine, prevalence of paraspinal abscess, and laboratory findings were not different between the GNB and GPC groups. The most common presenting symptom of patients with GNB pyogenic spondylitis was pain at the affected site, and 16.4% of patients presented with motor weakness. The lumbar spine was the most commonly involved site, and 48.4% of patients had paraspinal abscesses. Data regarding the clinical characteristics of pyogenic spondylitis caused by GNB are scarce. All 7 patients with pyogenic spondylitis caused by *E*. *coli* and the 10 patients caused by various GNB were > 63 years of age in previous studies [6,12]. However, in this study, the age distribution of the 62 patients with GNB pyogenic spondylitis was 37–85 years. In another study, patients with GNB pyogenic spondylitis had a lower prevalence of spinal epidural abscesses, paraspinal abscesses, WBC counts, and CRP values compared with methicillin-susceptible *S*. *aureus* (MSSA) pyogenic spondylitis [9]. These results differ from our findings and could have originated from different comparative groups since the previous study compared patients with pyogenic spondylitis caused by MSSA, while in the current work, patients with GPC including MSSA were used as a comparison. Furthermore, it has been reported that MSSA has a potent ability to form abscesses [13,14].

Multivariate analysis determined that the female gender, preexisting or synchronous genitourinary tract and intra-abdominal infections were associated with GNB infection in pyogenic spondylitis. Such an association is consistent with previous reports [7,9]. For the treatment of pyogenic spondylitis, empirical antibiotics are usually not recommended unless the patient is critically ill, neutropenic, or neurologically compromised [1]. The microbiological diagnosis of these infections is particularly important because long-term antibiotic therapy is required. A microbiological diagnosis was attempted using blood or tissue cultures. However, several studies have reported negative microbiological results in up to 30% of cases [15–17]. In this study, microorganisms were not identified in 24.1% of cases. In culture-negative infectious spondylitis, empirical antibiotics should be selected based on the clinical context. The suspicion of GNB as a cause of pyogenic spondylitis is important because inadequate empirical antibiotic treatments for GNB could be associated with neurologic complications [6]. Our study suggests that the use of antibiotics covering GNB, such as a third- or fourth-generation cephalosporin or carbapenem, should be considered when patients are female or have had a preexisting or synchronous genitourinary tract and intra-abdominal infection.

Identifying a potential source of infection, other than the spine, is crucial to the selection of empirical antibiotics to eradicate the infection. Preceding or synchronous infection at sites other than spine were reported in 30–50% of patients with pyogenic spondylitis [3,18]. The most commonly reported site of preceding infection was the genitourinary tract, followed by skin and soft tissues, respiratory tract, gastrointestinal tract, or oral cavity [11]. In our study, we identified extra-spinal infection in 28.5% of patients, and the most frequent infection site was the genitourinary tract, followed by skin and soft tissue, endocardium, intra-abdomen, and catheter. Pyogenic spondylitis caused by GNB was more frequently associated with genitourinary tract and intra-abdominal infections, and it could be surmised that GNB are the most common etiologic organisms of these infections. We determined that female gender was predominant in GNB infection, in contrast to other studies stating a fivefold male predominance in pyogenic spondylitis [19,20]. The predominance of females with GNB infection might be explained by a higher prevalence of urinary tract infection. In our study, the fluoroquinolone resistance rate in the *Enterobacteriaceae* family was >30%, and while not associated with a previous invasive spinal procedure, it was associated with increasing antimicrobial resistance in GNB globally [21].

When a diagnosis of pyogenic spondylitis is suspected clinically and radiologically, there is no universal recommendation for treatment. Some experts recommended a combination of levofloxacin and rifampin, or a combination of ciprofloxacin and clindamycin [1,22,23]. However, the existence of fluoroquinolone-resistant bacteria increases the risk of treatment failure and the selection of resistant mycobacteria in regions where tuberculosis remains prevalent. Further study of the risk factors associated with resistant organisms in pyogenic spondylitis is necessary.

Our study had several limitations. First, these data were collected retrospectively. Thus, unrecognized predisposing factors could have been distributed unequally between GPC and GNB pyogenic spondylitis. Second, this study included postoperative infection, as well as, healthcare-associated infection, even though the proportion of patients who had previous invasive spinal procedures, other than epidural block, was not different between the GNB and GPC groups. Therefore, the results of this study may not be truly indicative of community-onset pyogenic spondylitis.

In conclusion, female gender, preexisting or synchronous genitourinary tract, and intra-abdominal infection are predictive of GNB infection in pyogenic spondylitis.
